# Multidisciplinary care in Parkinson’s disease

**DOI:** 10.1007/s00702-024-02807-w

**Published:** 2024-07-23

**Authors:** David Weise, Inga Claus, Christian Dresel, Elke Kalbe, Inga Liepelt-Scarfone, Stefan Lorenzl, Christoph Redecker, Peter P. Urban

**Affiliations:** 1 Department of Neurology, Asklepios Fachklinikum Stadtroda, Stadtroda, Germany; 2 Department of Neurology, University of Leipzig, Leipzig, Germany; 3 Department of Neurology with Institute of Translational Neurology, University Hospital of Münster, 48149 Münster, Germany; 4 Department of Neurology, Section for Movement Disorders and Neurostimulation, Neuroimaging Center Mainz, Universitätsmedizin Mainz, Mainz, Germany; 5 Medical Psychology, Neuropsychology and Gender Studies, Center for Neuropsychological Diagnostics and Intervention (CeNDI), University Hospital Cologne and Medical Faculty, University of Cologne, Cologne, Germany; 6 German Center for Neurodegenerative Diseases (DZNE) and Hertie Institute for Clinical Brain Research, Department of Neurodegenerative Diseases, University of Tübingen, IB-Hochschule, Tübingen, Stuttgart, Germany; 7 Institute of Palliative Care, Paracelsus Medical University, Salzburg, Austria; 8 Department of Neurology, Department of Palliative Care, Hospital Agatharied, Hausham, Germany; 9 Clinic of Palliative Care, Ludwig Maximilians University, Munich, Germany; 10 Department of Neurology, Klinikum Lippe Lemgo, Rintelner Str. 85, D-32657 Lemgo, Germany; 11 Department of Neurology, Asklepios Klinik Barmbek, Hamburg, Germany

**Keywords:** Parkinson’s Disease, Multidisciplinary team, Non-pharmacological treatment, Multimodal complex treatment, Parkinson network

## Abstract

Parkinson’s Disease (PD) is a multifaceted and progressive disorder characterized by a diverse range of motor and non-motor symptoms. The complexity of PD necessitates a multidisciplinary approach to manage both motor symptoms, such as bradykinesia, gait disturbances and falls, and non-motor symptoms, including cognitive dysfunction, sleep disturbances, and mood disorders, which significantly affect patients’ quality of life. Pharmacotherapy, particularly dopaminergic replacement therapy, has advanced to alleviate many symptoms. However, these medications can also induce side effects or aggravate symptoms like hallucinations or orthostatic dysfunction, highlighting the need for comprehensive patient management. The optimal care for PD patients involves a team of specialists, including neurologists, physical and occupational therapists, speech-language pathologists, psychologists, and other medical professionals, to address the complex and individualized needs of each patient. Here, we illustrate the necessity of such a multidisciplinary approach in four illustrative PD cases with different disease stages and motor and non-motor complications. The patients were treated in different treatment settings (specialized outpatient clinic, day clinic, inpatient care including neurorehabilitation). The biggest challenge lies in organizing and implementing such comprehensive care effectively across different clinical settings.

## Introduction

**Fig. 1:**
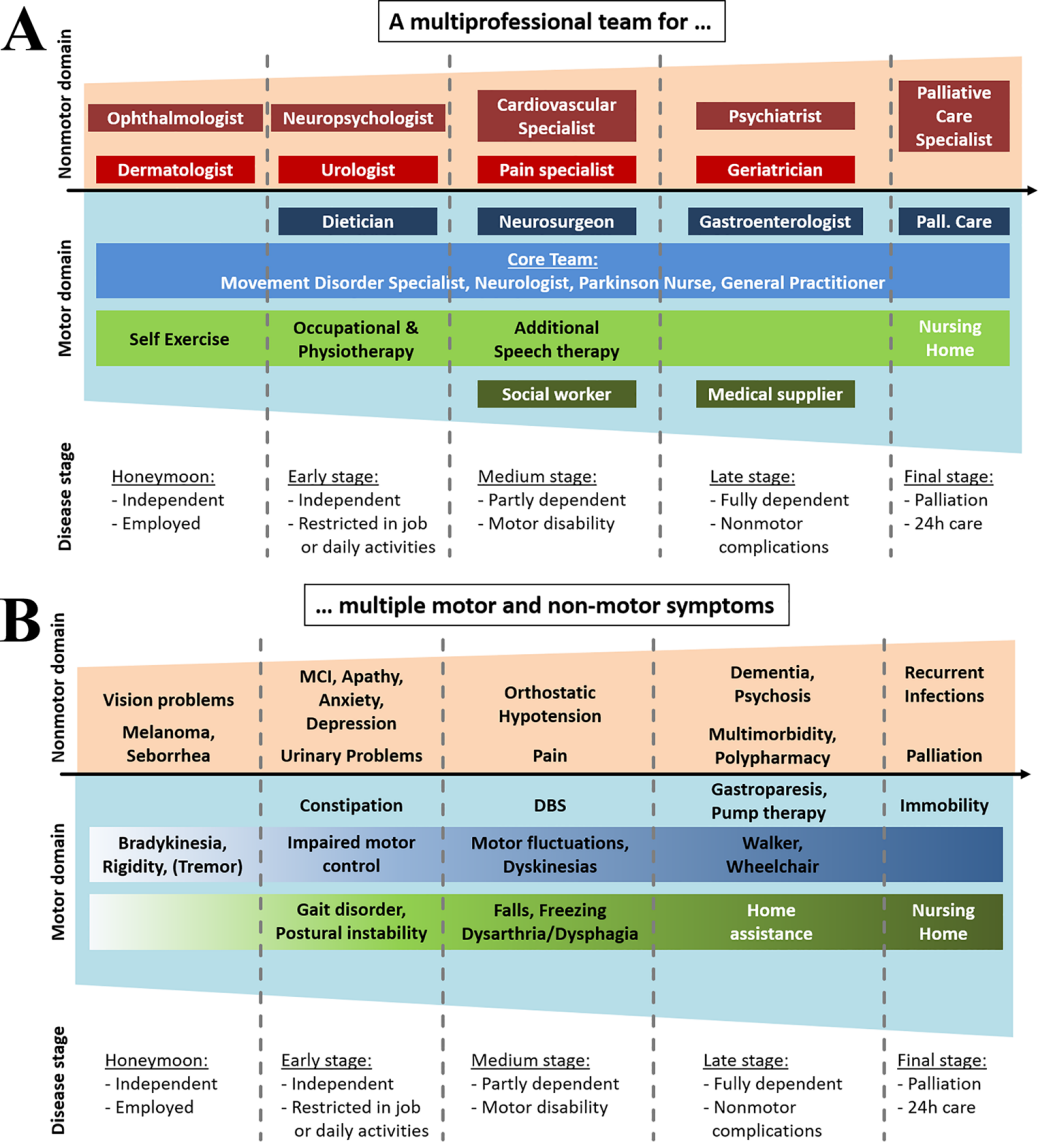
**A** Involvement of a multiprofessional team in the course of PD in order to manage **B** multiple motor and non-motor symptoms. *MCI* mild cognitive impairment

Parkinson’s Disease (PD) is a chronic, complex, progressive disorder with various motor and non-motor symptoms. The clinical phenotype is heterogeneous and the course of the disease varies notably between individual PD patients. Therefore, PD is rather a syndrome comprising different subtypes and probably different etiologies (Wüllner et al. 2023). The motor phenotypes are traditionally divided in the tremor-dominant, the equivalent and the akinetic-rigid typ, the later with earlier postural instability and gait difficulties (Jankovic et al. 1990). Later on, the importance of non-motor features of the disease were taken into greater account for subtyping PD using clinical and data-driven approaches (Marras and Lang 2013; Marras and Chaudhuri 2016; Sauerbier et al. 2017; Shakya et al. 2022). Quality of life of PD patients (and caregivers) is mainly driven by these non-motor symptoms, especially cognitive dysfunctions that become more and more evident in the course of the disease (Barone et al. 2009). However, sleep disturbances, autonomic symptoms or mood disorders but also psychological coping with the illness have a major impact on quality of life in PD (Poewe et al. 2017; Schapira et al. 2017). Pharmacotherapy, mainly based on dopaminergic replacement therapy, has developed over the last decades that improves motor and non-motor symptoms on the one side. Nevertheless, it may also worsen or lead to non-motor symptoms such as hallucinations, orthostatic dysfunction or impulse control disorder on the other side and does not cover all facets of PD and its symptoms. Due to this complexity of the disease and its treatment, it has become evident that PD patient’s management requires a multidisciplinary care to effectively manage and treat the individual motor and non-motor symptoms (Lidstone et al. 2020; Radder et al. 2019; Radder et al. 2020). Such a multidisciplinary care in PD involves a team-based approach to address the complex needs of patients. This team typically includes a neurologist and/or movement disorder specialist, the general practitioner, physical therapists, occupational therapists, speech-language pathologists, social workers as well as (neuro-)psychologists and/or psychiatrists (Table 1; Fig. 1; Qamar et al. 2017).

Other specialist physicians such as urologist, gastroenterologist, neuroophthalmologist, geriatrician and palliative care physician are also important members to the multiprofessional health care team and provide specialized care tailored to the individual patient’s needs and deficits (Radder et al. 2019; Radder et al. 2020).

**Table 1:** Key player of a multiprofessional team and their main tasks. *DBS* deep brain stimulation

Movement disorder specialist	- makes the right diagnosis - identifies the relevant motor and non-motor symptoms - manages PD by prescribing medications as well as overseeing treatment plans. However, in several countries or regions the generalist is the key player who deals regularly with the patient and coordinates the multidisciplinary treatment.
General practioner	- key player who deals regularly with the patient and coordinates the multidisciplinary treatment.
Parkinson Nurse	- supports both patients caregiver, offering clinical expertise, emotional support, and practical guidance throughout the disease’s progression - often serve as a liaison between different healthcare providers - may assist in managing advanced treatments (like DBS or infusion therapies)
Physical therapist	- improves mobility, balance, and strength through exercises tailored to the individual’s needs - refers to physicians about the motor- and non-motor symptoms to be addressed by drug treatment
Occupational therapist	- focusses on daily living activities, providing strategies and adaptations to help patients maintain independence
Speech-language pathologist	- address speech and swallowing difficulties providing exercises and techniques to improve communication and swallowing function.
Dietitian	- educate patients about the interactions between food and PD medications, provide strategies to alleviate gastrointestinal symptoms, offer education and support to patients and their caregivers
(Neuro-)Psychologist/Psychiatrist	- provide counselling, support and therapy for emotional issues such as depression, anxiety, or cognitive changes. - make the diagnosis of cognitive impairment especially at its early stages, when deficits are benign or need to differentiated from other behaviour symptoms such as depression or apathy - pre- (and post)operative neuropsychological diagnostic before DBS
Neurosurgical specialist	- involved when neurosurgical options such as deep brain stimulation may be considered, requiring collaboration with neurosurgeons and specialized nurses.
Social worker	- assists patients and their families in navigating the social and emotional challenges of living with PD, connecting them with support services and resources

*DBS* deep brain stimulation

The need of a multidisciplinary approach will be emphasized in four illustrative PD cases with different motor and non-motor subtypes treated in different clinical settings (overview in Table 2).

**Table 2:** Overview of the key complaints/symptoms in different disease stages treated by various members of the multidisciplinary team in different clinical settings in the four illustrative cases

Case *n*°	Disease stage	Primary/main complaints/symptoms	Involved team members	Type of care
1	Early stage PD	- Anxiety and depression - sexual dysfunction	- neuropsychologist, psychiatrist, psychotherapist - urologist	Specialized outpatient clinic
2	Mid stage PD	- Motor fluctuations and dyskinesia - gait disorder - slight dysphagia - constipation, nausea	- neurosurgeon - neuropsychologist - physiotherapist - speech therapist - dietitian	Day clinic
3	Mid stage PD	- axial deformation and pain - dysphagia with sialorrhia - orthostatic hypotension - optic hallucinations - mild cognitive impairment	- physiotherapist, occupational therapist, pain therapist - speech therapist - neurologist - neuropsychologist	Inpatient clinic
4	Late stage PD	- Gait disorder with freezing and falls - severe dysphagia - hallucinations - dementia	- physiotherapist, occupational therapist - speech therapist, palliative care specialist - gerontopsychiatrist - social worker	Inpatient clinic

## Illustrative PD cases

### Case 1

A male 55-year-old, married, came to the PD outpatient clinic of a university hospital. A suspected diagnosis of PD was made by the general practitioner eight months ago, but the patient had second thoughts about the PD diagnosis and assumed an orthopaedic problem instead. First symptoms occurred around two years earlier and comprised pain in the left shoulder and a fine motor disorder of the left hand. He further reported memory problems and difficulty in concentration, which had progressively worsened over the past five months. He also described that he was often very depressed and worried about his future. He was afraid that someone at work or in his circle of friends might find out about his illness. The denial of any problems leads to alienation from his wife. Besides, after a few special questions he complained about an urogenital dysfunction with subsequent loss of libido.

An extern MRI scan of the brain remained without pathological findings, but a dopamine transporter scintigraphy (DaTSCAN) showed bilateral, right-leading reduced dopamine transporter tracer binding.

At clinical examination, rigor and bradykinesia especially of the left body site as well as dysarthrophonia andhypomimia were found (MDS-UPRDS part III: 16 points). The PD medication consisted of rasagiline 1 mg and pramipexole ER 0.52 mg. A detailed evaluation of non-motor symptoms revealed a relevant hyposmia (Sniffin sticks examination 6/12), chronic constipation, and changes in mood and motivation. The patient mentioned fear of the PD diagnosis after all as well as of the symptoms associated with disease progression (i.e., “fear of progression”).

### Multidisciplinary evaluation and treatment

During his visit in the PD outpatient clinic the *movement disorder specialist* confirmed the PD diagnosis. Concerning the neurological part, a broad medical education with an extended overview especially about potentially helpful medication and further development of the disease was performed. The relevance of an active lifestyle especially including physical activity, mentally stimulating activities and a healthy diet was pointed out. Regarding to that, a recommendation for stepwise increase of the dose of pramipexole ER up to 1.57 mg.

A special *neuro-urological* assessment had been performed at a connected urological department. Herewith, a relevant, PD-associated erectile dysfunction was diagnosed. An urological medication with sildenafil was recommended as the patient showed no signs of orthostatic hypotension.

The patient was examined in detail by the *neuropsychologist*. The Montreal Cognitive Assessment (MoCA) score of 29 points indicated normal cognitive performance (cut-off value < 26 points, Dalrymple-Alford et al. 2010). Depressive mood and anxiety were assessed with the Beck Depression Inventory II (BDI II) and the Hospital Anxietxy and Depression Scale (HADS). Scores indicated moderate depressive symptoms (BDI II: 20 points; HADS: 12 points) and moderate anxiety (HADS: 15 points). The short form of the fear of progression questionnaire (FOP-Q) indicated high fear of progression (52 points; Folkerts et al. 2022). Therefore, medical pharmacotherapy with venlafaxine 75 mg was suggested by the *psychiatrist* with a possible combination of mirtazapine in case of persisting anxiety within the following month. Furthermore, cognitive behavioral therapy to treat depression and anxiety as well as physical interventions were recommended. As rehabilitation sport was not available close to home, the patient was encouraged to find some form of sports he liked and could imagine to conduct three times a week.

After 6 month, Mr. U. revealed a clear improvement of motor function (MDS-UPDRS III 8 points). Besides, depressive mood (BDI II: 14 point), anxiety (HADS: 9 points) and erectile dysfunction were ameliorated as well, with persisting problems in concentration especially at work. He reported that on the one hand, his family was shocked and burdened by the diagnosis; on the other hand, there was relief to know where the changes had come from. He had joined a Nordic Walking group and trained regularly.

### Case 2

A 67-year-old female patient was diagnosed with idiopathic PD six years ago. At that time, her symptoms included a considerable slowing of her gait, clumsiness and intermittent resting tremor of the right hand. In addition, the patient reported a REM sleep behavior disorder that had been present for about 10 years, symptoms of mild depression and constipation that had been present for years with about three defecations per week. She immediately benefited from the therapy with levodopa. She had had to discontinue additional treatment with pramipexole and ropinirole due to nausea and dizziness. The depressive symptoms had improved after diagnosis and start of dopaminergic treatment.

The patient currently reported end of dose fluctuations for at least one year (currently 400 mg levodopa per day in 4 single doses and an additional 100 mg ER at night). In addition, the constipation had increased. Defecation is painful and sometimes bloody. She usually suffers from nausea after meals.

Clinical examination revealed a pronounced right-sided akinetic-rigid syndrome with hypophonic dysarthria, hypomimia, bent forward gait, reduced arm swing on both sides and increased turning steps. Postural control was impaired.

### Multidisciplinary evaluation and treatment

The patient was admitted to a specialized day clinic for PD patients. During the visits, motor fluctuations with wearing off and some peak-dose dyskinesia became obvious. A transdermal rotigotine patch was added with increasing dose to 4 mg per day (in order to bypass the gastrointestinal tract) and levodopa medication was supplemented with the COMT inhibitor opicapone. *Neuropsychological* evaluation did not reveal relevant cognitive deficits (MoCA: 27 points). The patient received regular *physiotherapy* to improve her stride length and gait stability. Her mobility increased with dopaminergic therapy. Dysarthria was treated by the *speech therapist*, evaluation of swallowing revealed early signs of oro-pharyngeal dysphagia. For constipation, the patient was regularly given macrogol (polyethylene glycol) and the *dietitian* provided detailed nutritional advice. She should pay special attention to drinking 1.5–2.0 L of water regularly and include enough natural fiber in her daily meals. As this therapy and the increased physical activity already improved the stool frequency during the inpatient stay, drug treatment with prucalopride was not necessary. Due to improved, but still persisting, rather mild motor fluctuations the patient was informed in detail about advanced therapy options. As the patient was very interested and no obvious contraindications against deep brain stimulation were present an appointment in the *neurosurgery* outpatient clinic was organized. The patient was discharged after 15 days of treatment in the day clinic setting.

To improve therapy adherence in the home environment, the patient was contacted by telephone by a *PD nurse* four weeks after discharge and a few questions about taking medication in everyday life were clarified. The patient benefited from regular physical activity and was able to significantly increase her range of daily activities.

### Case 3

A 74-year-old man suffers from PD, starting with shoulder pain, decreased arm swing on the right side and micrographia. At the time of diagnosis 8 years ago hyposmia and rare events of REM sleep behaviour disorder had been reported. The pharmacological treatment started with rasagiline and pramipexol ER with increasing dosages (actually 2.1 mg/d). Three years ago, levodopa 300 mg/d was added.

Actually, the patient reported severe back pain since two months exaggerated by standing and walking together with some dullness and light-headedness all symptoms ceasing by lying in a recumbing position. He also suffered from sialorrhea during the day. The patient’s wife added that she had noticed a slight memory loss. He sometimes forgets to take his tablets regularly.

The patient presented with thoracolumbal camptocormia (35°), right sided pisa syndrome (10°), right dominant badykinesia and mild rigidity in the right arm. He walked slowly, taking short steps, and had decreased arm swing. Cognition was slightly impaired and he reported intermittent passage hallucinations about twice a month. Blood pressure was 120/65 mmHg when patient was lying down and the systolic blood pressure 80 to 90 mmHg while standing.

### Multidisciplinary evaluation and treatment

Due to the multiple manifestations of PD affecting activities of daily living the patient was included into an inpatient multimodal special treatment program (“Complex Parkinson therapy”) with pharmacological interventions and intensive ‘activating therapies’ (physiotherapy, occupational therapy, speech,-language-and swallowing therapy, physical exercise, art therapy). Neuropsychological evaluation revealed mild cognitive impairment (PD-MCI, Litvan et al. 2012), although the diagnostic criteria for Parkinson’s dementia were not met (Dubois et al. 2007). He further underwent cognitive training twice a week from the *occupational therapists*. Regarding camptocormia pramipexole ER and optic hallucinations was reduced to 1.05 mg/d and levodopa dosage increased to 500 mg/d. Together with concomitant intensive *physiotherapy* using active self-correction exercises with visual and proprioceptive feedback, passive and active trunk stabilization exercises and functional tasks camptocormia, Pisa syndrome and the walking pattern improved. However, as (the nociceptive) pain was not sufficiently alleviated pharmacological treatment with the low potent opioid tilidine with naloxone (50/4 mg twice a day) was added in consultation with the *pain therapist* resulting in sufficient pain relief. Metamizole and tramadol were avoided due to the risk of hypotension and anticholinergic effects, respectively.

Twenty-four-hour blood pressure profile showed a paradoxical increase of RR during the night to 160 to 170 mmHg (‘reverse dipper’). Therefore, an antihypertensive drug (candesartane 8 mg) in the evening was added and due to symptomatic orthostatic hypotension. The patient was instructed to sleep in a head-up position during the night and got compression stockings. Under these non-pharmacological interventions the orthostatic symptoms considerably improved, so that an additional pharmacological treatment during the day (e.g. midodrine) was not necessary.

Regarding sialorrhea the patient was treated by LSVT-loud by *speech therapist* and because anticholinergic treatment was not opportune due to passage hallucinations and mild cognitive impairment, the patient received ultrasound guided botulinum toxin injections bilaterally into the parotid and submandibular salivary glands (100 U Incobotulinumtoxin per side, Jost et al. 2019).

### Case 4

A 76-year-old patient with late-stage PD known for thirteen years was admitted to hospital from an assisted living home, to which he had been admitted four weeks previously. The reason for admission has been an acute deterioration in his condition with pneumonia and exsiccosis.

The PD medication consisted of levodopa 5 × 125 mg/d in combination with entacapone (5 × 200 mg) and rasagiline 1 mg. The patient was further treated with amitriptyline 50 mg for night.

### Multidisciplinary evaluation and treatment

Initially, the patient was somnolent, partially hallucinating and showed a leg-accentuated rigor, so that he was unable to walk and bedridden. He had a pronounced swallowing disorder as revealed by the *speech therapist*, which meant that a nasogastric tube would have been necessary to administer the medication. This initially was rejected by the relatives with reference to the existing advance directives in the form of a living will.

The opinion regarding life-prolonging measures was revised by the partner in the course of the now necessary discussion. She (and also the neurologist in charge) were of the opinion that he should be allowed to die after all. After intensive discussions together with the *palliative care specialist* in which it was explained that this would be a temporary measure, limited consent was obtained. The insertion of a permanent feeding tube was rejected in principle.

The patient’s usual medication for treating PD could be administered via the nasogastric tube (500 mg/d), in addition to intravenous administration of antibiotics and fluids. Due to the hallucinations (the anti-cholinergic acting drug) amitriptyline and later on entacapone were stopped. Within six days there was a pleasing improvement in the patient’s condition. The patient greeted us during the daily visits with his well-known sense of humour and showed no signs of sadness or a desire to die. His ability to walk with a walking aid also improved daily and the hallucinations became less frequent and occurred only in the later evening. Hence, after consulting a *gerontopsychiatrist* the neuroleptic drug quetiapine (50 mg/d) was added. During the subsequent neurorehabilitation with intensive physio-, occupational and speech therapy on our palliative care ward, the patient almost regained his pre-stationary condition. However, independence in activities of daily living was not attended why a nursing home was organized by the patient’s family after advice and support from the *social worker*.

## Discussion

These cases demonstrate that PD is heterogeneous, meaning it affects individuals differently in terms of symptoms (Fig. 1B), progression, and response to treatment. Therefore, a multidisciplinary approach is crucial for optimal treatment results in PD (Lidstone et al. 2020; Radder et al. 2019; Raddert al. 2020; Scherbaum et al. 2020; Ziegler et al. 2022). Multidisciplinary therapy ensures that patients receive care from a team of professionals with diverse expertise (comprehensive care; Table 1; Fig. 1A). Whereas neurologists, ideally specialists in movement disorders should have the overview of the diagnosis and course of the disease, physical therapists focus on improving mobility, balance, and strength through tailored exercise programs. Occupational therapists help patients maintain independence in daily activities by addressing fine motor skills, cognitive strategies and environmental modifications. Speech therapists assist with communication difficulties, swallowing problems, and voice control issues commonly seen in PD. Psychologists provide support for emotional well-being, helping patients cope with anxiety, depression, and adjustment to the disease as well as assess and treat cognitive dysfunctions (Hong et al. 2021; Orgeta et al. 2020; Pontone et al. 2021). Multidisciplinary teams conduct comprehensive assessments to understand each patient’s unique needs and challenges. Based on this assessment, they develop individualized treatment plans that may include a combination of medication adjustments, physical therapy exercises, speech therapy techniques, and cognitive strategies (tailored treatment plans). This personalized approach optimizes symptom management, enhances functional abilities and improves overall quality of life for PD patients (Eggers et al. 2018; Fabbri et al. 2022; Li et al. 2023).

However, PD impacts various aspects of life beyond motor symptoms. Patients often experience non-motor symptoms such as cognitive impairment, mood disturbances, sleep disturbances, and autonomic dysfunction (Barone et al. 2009). Additionally, PD can have significant psychosocial effects on patients and their families, including social isolation, caregiver stress, and financial strain (Macchi et al. 2020). Multidisciplinary teams address these holistic needs by providing comprehensive support services. This should involve psychological counselling, education about the disease and its management, support groups for patients and caregivers, assistance with navigating community resources, and strategies for optimizing quality of life (holistic support, Geerlings et al. 2023; Martinez-Martin et al. 2008; Thieken et al. 2022).

Multidisciplinary teams ensure continuity of care by coordinating efforts across various healthcare providers and settings. Regular follow-up appointments allow for assessment of treatment effectiveness, monitoring of disease progression, and adjustment of treatment plans as needed. This integrated approach minimizes treatment gaps, prevents complications, and maximizes functional independence and well-being for patients throughout the course of the disease ( Martinez-Martin et al. 2008; Rajan et al. 2020; van der Marck et al. 2013). Even in later stages of PD, when patients have new and different needs, disease management requires multidisciplinary treatment and palliative care (Fabbri et al. 2020; Macchi et al. 2020; Ortelli et al. 2018).

In summary, multidisciplinary therapy for PD offers comprehensive, personalized care that addresses the diverse needs of patients across physical, cognitive, emotional, and social domains. By integrating expertise from various healthcare professionals and providing holistic support, multidisciplinary teams optimize symptom management, enhance functional abilities, and improve overall quality of life for individuals living with PD.

The biggest challenge is how to organize and implement this inter- and multidisciplinary therapy.

### Concepts of inter- and multidisciplinary care of PD (in Germany)

There are different concepts of comprehensive multidisciplinary care, including access to neurologists, physical therapists, occupational therapists, speech therapists, neuropsychologists and other healthcare professionals specialized in PD management (Fig. 1A).

*Specialized outpatient care* (case 1) involves regular visits to neurologists or movement disorder specialists who have expertise in managing PD, largely available in urban conurbations (but less available in more rural areas). This setting is especially helpful in earlier disease stages. Visits in this setting may include medication management, symptom monitoring, and adjustments, as well as referrals to other specialists as needed (see other article in this issue). There are several advantages, but also limitations of outpatient clinic care for PD (Radder et al. 2020). Outpatient clinics provide individualized care tailored to the specific needs of each PD patient, including medication management, rehabilitation, and psychological support. They usually consist of a multidisciplinary team involving neurologists, nurses, therapists, and other healthcare professionals to address the diverse patients’ needs. It also offers greater accessibility and convenience for mobile and less affected patients, allowing them to receive treatment without the need for overnight stays or hospitalization. Furthermore, outpatient clinic care is often more cost-effective than inpatient care, reducing healthcare expenditures and making PD management more affordable for patients and healthcare systems (Steendam-Oldekamp and van Laar 2024; Steendam-Oldekamp et al. 2023; Yi et al. 2022; Zhang et al. 2022). Nevertheless, some patients may face challenges with transportation to outpatient clinics, impacting their ability to attend appointments regularly and receive timely care. Especially rural areas often have a shortage of neurologists, movement disorder specialists, and other healthcare professionals with expertise in PD management. Lack of reliable transportation options due limited public transportation, long travel distances, and inadequate infrastructure may hinder patients’ ability to attend medical appointments. Another disadvantage may be time constraints resulting in shorter appointment making it challenging to thoroughly address all concerns or provide comprehensive assessments, but also limited access to specialized equipment or diagnostic tests, potentially delaying certain aspects of care or necessitating referrals to other facilities. This is especially challenging to manage complex or severe cases of PD that require intensive interventions or close monitoring, but may even be feasible in an outpatient setting (Ortelli et al. 2018). Overall, a comprehensive inter-professional team hospital out-patient service may result in better quality of life compared to standard neurological services (Soh et al. 2016).

In *a day clinic setting* (case 2), a more profound diagnostic in more complex cases can be performed and patients typically receive a combination of medication management, physical, occupational, and possibly speech therapy or counseling. This setting is relatively new and still restricted to larger cities (such as Hamburg or Berlin). It is primarily intended for mid stage PD patients. A previous Cochrane Review still concluded that medical day hospital care for the elderly may be more effective than no intervention (but may have no clear advantage over other forms of comprehensive elderly medical services; Forster et al. 2008). However, this multidisciplinary day-clinic approach can support benefit on motor (Wade et al. 2003), but also on non-motor symptoms and QoL in PD-patients as shown by several other studies (Cohen et al. 2021; Fründt et al. 2018, 2020; Krause et al. 2022). Day clinic care allows for intensive treatment and therapy sessions to be delivered in a condensed timeframe, typically within a single day or several consecutive days. This can lead to more rapid improvements in symptoms and functional abilities (Ellis et al. 2008; Krause et al. 2022). Compared to inpatient care (see below) day clinic care minimizes disruption to the patient’s daily routine, as patients can return home at the end of each day. This allows them to maintain their independence and continue with their usual activities outside of clinic hours on the one side, but keeps the opportunity for social support and peer interaction among PD patients attending therapy sessions together on the other side. This social aspect can enhance motivation, morale, and overall well-being.

However, someone has to keep in mind that treatment sessions may be limited in duration due to time constraints in day clinics. This could result in less time for individualized therapy or comprehensive assessment compared to longer inpatient stays (Cohen et al. 2023). Unlike inpatient care settings where patients are continuously monitored, day clinic care may offer limited access to ongoing monitoring and supervision, potentially delaying the detection of complications or adverse events. As it is the case for specialized outpatient care, patients may face transportation and logistical challenges, especially if they live far from the clinic or require assistance with mobility. Last but not least, day clinics are not widely available and restricted to conurbations.

*Inpatient care* (care 3 and 4) involves a more intensive approach, often necessary for patients with more advanced PD, immobile patients or those experiencing complications such as medication-related side effects, falls, or cognitive decline. Specialized inpatient PD care is largely available in Germany in University hospitals but also specialized movement disorder clinics. Multimodal complex treatment may include medication adjustments, deep brain stimulation (DBS) surgery, initiation of continuously infused drugs (levodopa, foslevodopa, apomorphine), specialized nursing care, and intensive rehabilitation services (Radder et al. 2020). The obvious advantage of inpatient care is that it allows for continuous monitoring and supervision by healthcare professionals, providing prompt intervention for any complications or changes in the patient’s condition. This setting facilitates the delivery of multimodal complex treatmentwithin a controlled hospital environment and structured therapy sessions as shown in several studies (Ellis et al. 2008; Ferrazzoli et al. 2018; Hartelt et al. 2020; Heimrich and Prell 2021; Scherbaum et al. 2020; Scherbaum et al. 2022; Steendam-Oldekamp and van Laar 2024; Ziegler et al. 2022). Not to be underestimated are the opportunities for psychosocial support and counseling for PD patients and their families, addressing the emotional, social, and psychological aspects of living with PD (Geerlings et al. 2023; Martinez-Martin et al. 2008). In less acute cases, where medication adjustments or advanced therapies are less necessary, PD patients can also be treated in specialized Parkinson’s rehabilitation clinics focused on non-pharmacological therapies.

However, inpatient care often requires patients to stay in the hospital for an extended period, leading to a loss of independence and disruption to their daily routines. Inpatient settings also carry a higher risk of hospital-acquired infections, particularly for vulnerable populations such as elderly PD patients. Additionally, transitioning from inpatient to outpatient care settings can pose challenges for PD patients, including adjustments to new medication regimens, rehabilitation plans, and coordination of follow-up appointments. Finally yet importantly, inpatient care is generally more expensive than outpatient or day clinic care due to the costs associated with hospitalization, specialized services, and round-the-clock nursing care (Johnson et al. 2013; Steendam-Oldekamp and van Laar 2024; Steendam-Oldekamp et al. 2023; Yi et al. 2022; Zhang et al. 2022. 7).

Up to now, there are no studies comparing clinical, motor and non-motor outcomes between these different treatment settings. Balancing all these advantages and disadvantages is essential to ensure optimal care for each individual PD patients in different clinic settings. One opportunity may offer the treatment with a network (Bloem et al. 2020; Nijkrake et al. 2010; van Munster et al. 2020). Treating patients within a *PD network* involves a collaborative approach that integrates various healthcare providers, support services, and (in- and outpatient) resources to deliver comprehensive care tailored to the individual needs of PD patients (Lummer et al. 2024). A PD network typically involves a multidisciplinary team of healthcare professionals, including neurologists, movement disorder specialists, nurses, physical therapists, occupational therapists, speech therapists, psychologists, and social workers. This team collaborates to address the diverse needs of PD patients and provide holistic care ( Prell et al. 2020). These networks prioritize early diagnosis and intervention to optimize patient outcomes. This may involve specialized (in- and/or outpatient) clinics or centers that offer timely assessments, diagnostic evaluations, and treatment initiation, enabling proactive management of PD symptoms. A strong argument for PD networks is that they emphasize patient education and empowerment to promote active participation in disease management. Patients and their caregivers receive information about PD, treatment options, symptom management strategies, and lifestyle modifications to enhance self-management skills and improve quality of life (Tennigkeit et al. 2020). PD networks even leverage telemedicine and remote monitoring technologies to facilitate access to care, especially for patients in rural or underserved areas (Prell et al. 2020; van de Warrenburg et al. 2021).

Ideally, the generalist, the primary care physician, is part of the core team. They play a pivotal role in the initial recognition and diagnosis of PD based on clinical symptoms and history (Baldin et al. 2020). While they may not provide definitive diagnosis, they can refer patients to neurologists or movement disorder specialists for further evaluation and confirmation. Furthermore, generalists monitor together with the neurologist PD patients’ overall medication and potential interaction especially in the context of polypharmacy. Depending on the neurological skills, generalists may adjust dosages in a certain range based on symptom severity and response. They coordinate care for PD patients, facilitating communication between specialists, therapists, and other healthcare providers involved in the patient’s treatment plan. They ensure seamless transitions between different levels of care and address any healthcare needs beyond PD management (Plouvier et al. 2017).

In summary, multidisciplinary care is crucial for PD that affect various motor and non-motor aspects of a person’s life. This approach involves a team of healthcare professionals like generalists, neurologists (and/or ideally movement disorder specialist), physiotherapists, speech therapists, psychologists, and social workers, who work together to address different aspects of the disease, from motor symptoms to emotional well-being and quality of life. This comprehensive approach ensures tailored, patient-centred treatment plans and better outcomes for patients. These multiprofessional needs can be addressed in different out- and inpatient conditions dependent on patients’ disease stage, motor and non-motor complications, comorbidities and mobility.

## References

[CR2] Baldin et al (2020) Low cost screening for features of Prodromal Parkinson’s Disease in General Medical Practice in Italy. J Parkinsons Dis 10(2):711–715 32176656 10.3233/JPD-191868

[CR1] Barone P et al (2009) The PRIAMO study: a multicenter assessment of nonmotor symptoms and their impact on quality of life in Parkinson’s disease. Mov Disord 24:1641–1649 19514014 10.1002/mds.22643

[CR3] Bloem BR et al (2020) Integrated and patient-centred management of Parkinson’s disease: a network model for reshaping chronic neurological care. Lancet Neurol 19:623–634 32464101 10.1016/S1474-4422(20)30064-8 PMC9671491

[CR4] Cohen et al (2021) Multidisciplinary intensive outpatient rehabilitation program for patients with moderate-to-advanced Parkinson’s disease. NeuroRehabilitation 49(1):47–55 33998554 10.3233/NRE-210031

[CR5] Cohen M, Herman T, Ganz N, Badichi I, Gurevich T, Hausdorff JM (2023) Multidisciplinary intensive Rehabilitation Program for people with Parkinson’s Disease: gaps between the Clinic and Real-World mobility. Int J Environ Res Public Health 21(5):3806 10.3390/ijerph20053806 PMC10001519 36900826

[CR6] Dalrymple-Alford JC et al (2010) The MoCA: well-suited screen for cognitive impairment in Parkinson disease. Neurology 9(19):1717–1725 10.1212/WNL.0b013e3181fc29c9 21060094

[CR7] Dubois B et al (2007) Diagnostic procedures for Parkinson’s disease dementia: recommendations from the movement disorder society task force. Mov Disord 22(16):2314–2324 18098298 10.1002/mds.21844

[CR8] Eggers C, Dano R, Schill J, Fink GR, Hellmich M, Timmermann L, CPN study group (2018) Patient-centered integrated healthcare improves quality of life in Parkinson’s disease patients: a randomized controlled trial. J Neurol 265(4):764–773 29392459 10.1007/s00415-018-8761-7

[CR9] Ellis T, Katz DI, White DK, DePiero TJ, Hohler AD, Saint-Hilaire M (2008) Effectiveness of an inpatient multidisciplinary rehabilitation program for people with Parkinson disease. Phys Ther 88(7):812–819 18436568 10.2522/ptj.20070265

[CR11] Fabbri M, Kauppila LA, Ferreira JJ, Rascol O (2020) Challenges and perspectives in the management of late-stage Parkinson’s Disease. J Parkinsons Dis 10(s1):S75–S83 32568114 10.3233/JPD-202096 PMC7592689

[CR10] Fabbri M, Coelho M, Garon M, Biundo R, Mestre TA, Antonini A (2022) Personalized care in late-stage Parkinson’s Disease: challenges and opportunities. On behalf of iCARE-Pd Consortium. J Pers Med 18(5):813 10.3390/jpm12050813 PMC9147917 35629235

[CR12] Ferrazzoli D et al (2018) Efficacy of intensive multidisciplinary rehabilitation in Parkinson’s disease: a randomised controlled study. J Neurol Neurosurg Psychiatry 89:828–835 29321141 10.1136/jnnp-2017-316437 PMC6204945

[CR13] Folkerts AK et al (2022) Fear of Progression is determined by anxiety and self-efficacy but not Disease-Specific parameters in patients with Parkinson’s Disease: Preliminary Data from a Multicenter cross-sectional study. J Parkinsons Dis 12(8):2543–2553 36189603 10.3233/JPD-223314

[CR14] Forster A, Young J, Lambley R, Langhorne P (2008) Medical day hospital care for the elderly versus alternative forms of care. Cochrane Database Syst Rev 8(4):CD001730 10.1002/14651858.CD001730 10796660

[CR15] Fründt O et al (2018) The Hamburg Parkinson day-clinic: a new treatment concept at the border of in- and outpatient care. J Neural Transm (Vienna) 125(10):1461–1472 30167934 10.1007/s00702-018-1918-9

[CR16] Fründt O, Veliqi E, Schönwald B, Sychla P, Gerloff C, Buhmann C (2020) The Hamburg Parkinson day-clinic: a new treatment concept at the border of in- and outpatient care. Fortschr Neurol Psychiatr 88(6):362–373 32303075 10.1055/a-1083-6316

[CR17] Geerlings AD et al (2023) Caregiver burden in Parkinson’s disease: a mixed-methods study. BMC, Med 10;21(1):247 10.1186/s12916-023-02933-4 PMC10332089 37424022

[CR19] Hartelt E, Scherbaum R, Kinkel M, Gold R, Muhlack S, Tönges L (2020) Parkinson’s disease multimodal complex treatment (PD-MCT): analysis of therapeutic effects and predictors for improvement. J Clin Med 9(6):1874 32560079 10.3390/jcm9061874 PMC7356837

[CR20] Heimrich KG, Prell T (2021) Short- and long-term effect of Parkinson’s Disease Multimodal Complex Treatment. Brain Sci 3(11):1460 10.3390/brainsci11111460 PMC8615811 34827459

[CR21] Hong CT, Tan S, Huang TW (2021) Psychotherapy for the treatment of anxiety and depression in patients with Parkinson Disease: a Meta-analysis of Randomized controlled trials. J Am Med Dir Assoc 22(11):2289–2295e2 33957132 10.1016/j.jamda.2021.03.031

[CR22] Jankovic J et al (1990) Variable expression of Parkinson’s disease: a base-line analysis of the DATATOP cohort. The Parkinson Study Group. Neurology 40(10):1529–1534 2215943 10.1212/wnl.40.10.1529

[CR23] Johnson SJ et al (2013) Costs of Parkinson’s disease in a privately insured population. PharmacoEconomics 31(9):799–806 23907717 10.1007/s40273-013-0075-0 PMC3757266

[CR24] Jost WH et al (2019) SIAXI: Placebo-controlled, randomized, double-blind study of incobotulinumtoxinA for sialorrhea. Neurology 23(17):e1982–e1991 10.1212/WNL.0000000000007368 PMC6511076 30918101

[CR25] Krause P, Berking S, Astalosch M, Grünheid R, Kühn AA (2022) Motor and non-motor improvements following short-term multidisciplinary day-clinic care in parkinsons disease. J Neural Transm (Vienna) 129(12):1419–1426 36335542 10.1007/s00702-022-02562-w PMC9649470

[CR26] Li T, Zou X, Kang Y, Sun M, Huang X, Duan X (2023) A meta-analysis of the effect of multidisciplinary comprehensive care on health-related quality of life and unified Parkinson’s Disease Rating Scale in Parkinson’s disease. Adv Clin Exp Med 32(6):623–631 36920262 10.17219/acem/157241

[CR27] Lidstone SC, Bayley M, Lang AE (2020) The evidence for multidisciplinary care in Parkinson’s disease. Expert Rev Neurother 20(6):539–549 32479209 10.1080/14737175.2020.1771184

[CR28] Litvan I et al (2012) Diagnostic criteria for mild cognitive impairment in Parkinson’s disease: Movement Disorder Society Task Force guidelines. Mov Disord 27(3):349–356 22275317 10.1002/mds.24893 PMC3641655

[CR29] Lummer C, Eggers C, Becker A, Demandt F, Warnecke T, Parkinson Netzwerke Deutschland e.V (2024) Interdisciplinary network care collaboration in Parkinson’s disease: a baseline evaluation in Germany. Neurol Res Pract 11(1):5 10.1186/s42466-023-00300-5 PMC10782567 38200604

[CR30] Macchi ZA et al (2020) Patient and caregiver characteristics associated with caregiver burden in Parkinson’s disease: a palliative care approach. Ann Palliat Med 9(Suppl 1):S24–S33 31735048 10.21037/apm.2019.10.01

[CR31] Marras C, Chaudhuri KR (2016) Nonmotor features of Parkinson’s disease subtypes. Mov Disord 31(8):1095–1102 26861861 10.1002/mds.26510

[CR32] Marras C, Lang A (2013) Parkinson’s disease subtypes: lost in translation? J Neurol Neurosurg Psychiatry 84(4):409–415 22952329 10.1136/jnnp-2012-303455

[CR33] Martinez-Martin P, Arroyo S, Rojo-Abuin JM, Rodriguez-Blazquez C, Frades-Payo B, Paz S (2008) Burden, perceived health status, and mood among caregivers of PD patients. Mov Disord 23(12):1673–1680 18709684 10.1002/mds.22106

[CR34] Nijkrake MJ et al (2010) The ParkinsonNet concept: development, implementation and initial experience. Mov Disord 15(7):823–829 10.1002/mds.22813 20461798

[CR35] Orgeta V, McDonald KR, Poliakoff E, Hindle JV, Clare L, Leroi I (2020) Cognitive training interventions for dementia and mild cognitive impairment in Parkinson’s disease. Cochrane Database Syst Rev 26(2):CD011961 10.1002/14651858.CD011961.pub2 PMC7043362 32101639

[CR36] Ortelli P et al (2018) Effectiveness of a goal-based Intensive Rehabilitation in Parkinsonian patients in Advanced stages of Disease. J Parkinsons Dis 8(1):113–119 29480227 10.3233/JPD-171247

[CR37] Plouvier AOA, Olde Hartman TC, Verhulst CEM, Bloem BR, van Weel C, Lagro-Janssen ALM (2017) Parkinson’s disease: patient and general practitioner perspectives on the role of primary care. Fam Pract 1(2):227–233 10.1093/fampra/cmw115 28419289

[CR38] Poewe W et al (2017) Parkinson disease. Nat Rev Dis Primers 23:3:17013 10.1038/nrdp.2017.13 28332488

[CR39] Pontone GM, Mills KA (2021) Optimal treatment of depression and anxiety in Parkinson’s Disease. Am J Geriatr Psychiatry 29(6):530–540 33648830 10.1016/j.jagp.2021.02.037

[CR40] Prell T et al (2020) Recommendations for Standards of Network Care for patients with Parkinson’s Disease in Germany. J Clin Med 13(5):1455 10.3390/jcm9051455 PMC7290836 32414071

[CR41] Qamar MA, Harington G, Trump S, Johnson J, Roberts F, Frost E (2017) Multidisciplinary care in Parkinson’s Disease. Int Rev Neurobiol 132:511–523 28554420 10.1016/bs.irn.2017.02.001

[CR43] Radder DLM et al (2020) Recommendations for the Organization of Multidisciplinary Clinical Care Teams in Parkinson’s Disease. J Parkinsons Dis 10(3):1087–1098 32444563 10.3233/JPD-202078 PMC7415700

[CR42] RadderDLM et al (2019) Multidisciplinary care for people with Parkinson’s disease: the new kids on the block! Expert Rev Neurother 19(2):145–157 30570362 10.1080/14737175.2019.1561285

[CR44] Rajan R et al (2020) Integrated Care in Parkinson’s Disease: a systematic review and Meta-analysis. Mov Disord 35(9):1509–1531 32598094 10.1002/mds.28097

[CR45] Sauerbier A, Rosa-Grilo M, Qamar MA, Chaudhuri KR (2017) Nonmotor Subtyping in Parkinson’s Disease. Int Rev Neurobiol 133:447–478 28802928 10.1016/bs.irn.2017.05.011

[CR46] Schapira AHV, Chaudhuri KR, Jenner P (2017) Non-motor features of Parkinson disease. Nat Rev Neurosci 18(7):435–450 28592904 10.1038/nrn.2017.62

[CR47] Scherbaum R et al (2020) Parkinson’s Disease Multimodal Complex Treatment improves motor symptoms, depression and quality of life. J Neurol 267(4):954–965 31797086 10.1007/s00415-019-09657-7

[CR48] Scherbaum R et al (2022) Parkinson’s disease multimodal complex treatment improves gait performance: an exploratory wearable digital device-supported study. J Neurol 269(11):6067–6085 35864214 10.1007/s00415-022-11257-x PMC9553759

[CR49] Shakya S, Prevett J, Xiao H, Ran X (2022) Characterization of Parkinson’s Disease subtypes and related attributes. Front Neurol 23:13810038 10.3389/fneur.2022.810038 PMC9167933 35677337

[CR50] Soh SE, Morris ME, Watts JJ, McGinley JL, Iansek R (2016) Health-related quality of life in people with Parkinson’s disease receiving comprehensive care. Aust Health Rev 40(6):613–618 26910356 10.1071/AH15113

[CR51] Steendam-Oldekamp E, van Laar T (2024) The effectiveness of Inpatient Rehabilitation in Parkinson’s Disease: a systematic review of recent studies. J Parkinsons Dis 1:1–20 10.3233/JPD-230271 PMC11380234 38788087

[CR52] Steendam-Oldekamp E, Weerkamp N, Vonk JM, Bloem BR, van Laar T (2023) Combined multidisciplinary in/outpatient rehabilitation delays definite nursing home admission in advanced Parkinson’s disease patients. Front Neurol 14:1128891 37122300 10.3389/fneur.2023.1128891 PMC10133548

[CR53] Tennigkeit J et al (2020) Structured care and self-management education for persons with Parkinson’s Disease: why the First does not go without the second-systematic review, experiences and implementation concepts from Sweden and Germany. J Clin Med 28(9):2787 10.3390/jcm9092787 PMC7563525 32872258

[CR54] Thieken F et al (2022) Development of a Multidimensional Assessment Tool for the Evaluation of Holistic Quality of Life in Parkinson’s Disease. J Parkinsons Dis 12(1):361–370 34602498 10.3233/JPD-202391

[CR56] van de Warrenburg BP, Tiemessen M, Munneke M, Bloem BR (2021) The Architecture of Contemporary Care Networks for Rare Movement disorders: leveraging the ParkinsonNet experience. Front Neurol 30:12:638853 10.3389/fneur.2021.638853 PMC8042326 33859608

[CR55] van der Marck MA et al (2013) Integrated multidisciplinary care in Parkinson’s disease: a non-randomised, controlled trial (IMPACT). Lancet Neurol 12:947–956 23988337 10.1016/S1474-4422(13)70196-0

[CR57] van Munster M, Tönges L, Loewenbrück KF, Warnecke T, Eggers C (2020) Building a Parkinson-Network-Experiences from Germany. J Clin Med 2020 25;9(9):2743 10.3390/jcm9092743 PMC7563415 32854328

[CR58] Wade DT, Gage H, Owen C, Trend P, Grossmith C, Kaye J (2003) Multidisciplinary rehabilitation for people with Parkinson’s disease: a randomised controlled study. J Neurol Neurosurg Psychiatry 74(2):158–162 12531939 10.1136/jnnp.74.2.158 PMC1738276

[CR59] Wüllner U et al (2023) The heterogeneity of Parkinson’s disease. J Neural Transm (Vienna) 130(6):827–838 37169935 10.1007/s00702-023-02635-4 PMC10174621

[CR60] Yi ZM, Li XY, Wang YB, Wang RL, Ma QC, Zhao RS, Chen LC (2022) Evaluating the direct medical cost, drug utilization and expenditure for managing Parkinson’s disease: a costing study at a medical center in China. Ann Transl Med 10(6):330 35433954 10.21037/atm-22-1014 PMC9011260

[CR61] Zhang H, Zhou W, Zhang D (2022) Direct Medical costs of Parkinson’s Disease in Southern China: a cross-sectional study based on Health Insurance Claims Data in Guangzhou City. Int J Environ Res Public Health 9(6):3238 10.3390/ijerph19063238 PMC8953775 35328925

[CR62] Ziegler K et al (2022) Activities of daily living are improved by inpatient multimodal complex treatment for PD—a real-world cohort study. Mov Disord Clin Pract 7(1):42–54 10.1002/mdc3.13578 PMC9847313 36698998

